# Disparities in Atrial Fibrillation Metabolic Risk in Indonesia: Time for Sociodemographic Targeted Interventions

**DOI:** 10.1016/j.jacasi.2026.03.005

**Published:** 2026-05-05

**Authors:** Ardian Rizal, Mohammad Saifur Rohman, Fatchiyah Fatchiyah, Hidayat Sujuti, Irzal Rakhmadhani, Veny Mayangsari, Muhammad Isra Rafidin Rayyan

**Affiliations:** a Doctoral Program in Medical Science, Faculty of Medicine, Universitas Brawijaya, Malang, East Java, Indonesia; b Department of Cardiology and Vascular Medicine, Faculty of Medicine, Universitas Brawijaya, Malang, Indonesia; c Research Centre of Smart Molecule of Natural Resource, Universitas Brawijaya; d Department of Ophthalmology, Faculty of Medicine, Universitas Brawijaya/Dr Saiful Anwar General Hospital Malang, East Java, Indonesia; e Faculty of Medicine, Universitas Jember, Jember, East Java, Indonesia

**Keywords:** epidemiology, health disparity, metabolic syndrome, mortality, population attributable fraction, socio-demographic index

Atrial fibrillation (AF) poses a significant challenge to health care systems, driven by aging populations and an increasing number of metabolic risk factors.^1^^,^^2^ The latest European Society of Cardiology guidelines emphasize comorbidity and risk-factor control (“C”) as a pivotal management strategy.^3^

Indonesia, the world’s largest archipelagic state and fourth most populous country, presents a complex socio-demographic landscape. The profile of metabolic risk is believed to be heterogeneous across its provinces.

Socio-demographic index (SDI) is a composite measure of income, education, and fertility. It serves as a robust tool to capture social and economic conditions.^4^ Rather than relying on average national data, stratifying by SDI can reveal patterns in disease risk.

This study aimed to quantify the population attributable fraction (PAF) of key metabolic risk factors (hypertension, diabetes mellitus [DM], and obesity) across Indonesia’s provinces from 2000 to 2021, providing evidence for the design of SDI-targeted interventions to address nationwide disparities.

## Methods

### Data source and study design

The authors analyzed publicly accessible data from the Global Burden of Disease (GBD) 2000-2021 database, which included 34 Indonesian provinces.^5^ We examined metabolic risk factors of AF: hypertension, DM, and obesity. We classified provinces into low-, middle-, and high-SDI groups based on their baseline SDI tertile in 2000. This study used de-identified, publicly available secondary data from the GBD database; therefore, it was deemed exempt from formal institutional review board approval. This study complies with the Guidelines for Accurate and Transparent Health Estimates Reporting (GATHER) statement.

### PAF

Represents the proportion of disease burden (measured by prevalence, deaths, and disability-adjusted life years [DALYs]) that can be reduced if exposure were lowered to its lowest possible level of risk. For hypertension and obesity, the estimation of PAF was extracted directly from the GBD. Because the PAF value for AF in DM was not available in the GBD, we used the following equation.^6^

$$PAF=\frac{P\left(RR-1\right)}{P\left(RR-1\right)+1}$$

Where P is the population exposure proportion (data extracted from GBD), and RR is the relative risk for AF per exposure category taken from a recent meta-analysis.^7^

### Estimated annual percentage change

We used the estimated annual percentage change (EAPC) to examine long-term trends in mortality, DALYs, and the prevalence of AF. We derived the EAPC from a log-linear regression analysis of age-standardized rates:

$$EAPC\;\left(\%\right)=100\times \left(e^{\beta }-1\right)$$

Where β is the regression coefficient of the year on the natural log of the rate.

## Results

### National trends of metabolic risk burden

**Figure 1:**
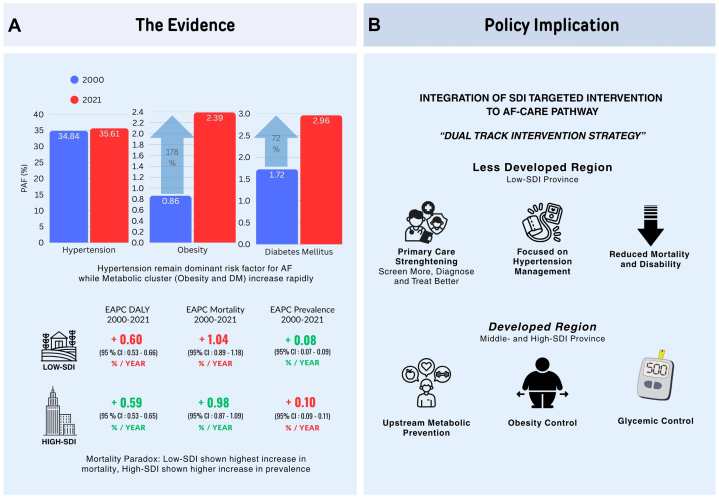
Metabolic Risk Drivers and Policy Pathways for AF (A) Changes in population attributable fractions for atrial fibrillation (AF) risk factors between 2000 and 2021. Hypertension remains the leading contributor, whereas obesity and diabetes mellitus (DM) show the largest proportional increases, indicating a growing “metabolic cluster.” Estimated annual percentage change (EAPC) metrics highlight divergent socio-demographic index (SDI) trajectories: low-SDI areas experience the fastest rise in AF mortality and disability despite smaller metabolic risk growth, whereas high-SDI areas show greater increases in AF prevalence. (B) These patterns are translated into a dual-track strategy integrated into AF care pathways: strengthening primary care, screening, diagnosis, and blood pressure management in less-developed regions, and upstream metabolic prevention emphasizing obesity and glycemic control in developed regions to reduce mortality and disability.

Hypertension remained the leading metabolic risk factor for AF burden throughout 2000-2021, with PAF increasing slightly from 34.84% (95% CI: 12.88-54.96) to 35.61% (95% CI: 12.72-55.77) (+0.77 pp; +2.21%). DM PAF increased from 1.72% (95% CI: 1.54-1.91) to 2.96% (95% CI: 2.65-3.29) (+1.24 pp; +72.16%), whereas obesity increased from 0.86% (95% CI: 0.25-1.73) to 2.39% (95% CI: 0.99-4.30) (+1.53 pp; +178.90%), establishing obesity and DM as the fastest growing contributors (Figure 1A).

### SDI stratified trends in AF burden

The DALYs burden increased fastest in the low-SDI group (EAPC: 0.60%; 95% CI: 0.53-0.66), followed by high-SDI (EAPC: 0.59%; 95% CI: 0.53-0.65) and middle-SDI (EAPC: 0.41%; 95% CI: 0.34-0.49). Mortality followed a similar pattern: low-SDI (EAPC: 1.04%; 95% CI: 0.89-1.18), high-SDI (EAPC: 0.98%; 95% CI: 0.87-1.09), and middle-SDI (EAPC: 0.67%; 95% CI: 0.51-0.83). In contrast, prevalence increased most rapidly in the high-SDI group (EAPC: 0.10%; 95% CI: 0.09-0.11), with slower increases in low-SDI (EAPC: 0.08%; 95% CI: 0.07-0.09) and middle-SDI (EAPC: 0.07%; 95% CI: 0.06-0.08) (Figure 1A).

### SDI-stratified differences in metabolic risk burden

Metabolic risk patterns varied by exposure. Hypertension PAF showed no significant difference between groups (global *P* = 0.475), with comparable medians across SDI (high: 33.38%; Q1-Q3: 33.2%-35.5%; middle: 35.37%; Q1-Q3: 33.7%-35.6%; and low: 34.75%; Q1-Q3: 34.5%-35.2%). Obesity showed a clear SDI gradient (*P* < 0.001). The highest PAF was observed in high-SDI (mean: 4.91% ± 0.54%), intermediate in middle-SDI (mean: 3.02% ± 0.93%), and lowest in low-SDI (mean: 2.44% ± 1.02%). DM PAF showed no stratified differences (global *P* = 0.747), with medians nearly identical across groups.

## Discussion

This study provides the first comprehensive analysis of the metabolic PAF for AF in Indonesia, revealing that the drivers of AF burden are substantial but unevenly distributed. Aligning with global data, hypertension is the most important modifiable risk for AF development.^3^^,^^8^ Given that less than half of Indonesians with hypertension meet the target blood pressure, largely due to high sodium diet habits and low adherence to medication.^9^ Therefore, controlling hypertension remains a priority for the AF prevention program.

However, metabolic drivers track closely with socioeconomic development, suggesting that national averages are insufficient for “one-size-fits-all” policies. DM and obesity are emerging rapidly as additional drivers, reflecting ongoing epidemiologic and nutritional transitions. This shift is most pronounced in developed, urbanized provinces, where metabolic risk clustering accompanies modernization and related lifestyle changes.

Less-developed provinces exhibit a mortality paradox: despite a slower increase in prevalence, adverse outcomes (disability and mortality) are worsening more rapidly than in developed regions. This suggests health system capacity constraints, including limited detection of new cases, delayed diagnosis, suboptimal management, and limited access to essential drugs.^10^

### Policy implications

This finding supports SDI-targeted intervention integrated into the AF-CARE clinical framework pathway. We propose a dual-track intervention strategy (Figure 1B). In the less-developed region, it is necessary to prioritize primary care strengthening and optimizing hypertension control to address rising mortality. In the developed region, it is necessary to focus on upstream metabolic prevention through weight and glycemic management.

Integrating these SDI-stratified approaches into Indonesia's health policy could translate these epidemiologic insights into an equitable population-level impact.

### Study limitations

This study has limitations inherent to the GBD methodology. As an ecological analysis, findings reflect population-level associations and cannot definitively establish causality at the individual level. Furthermore, PAF estimates rely on theoretical minimum risk levels that may vary across local contexts. Finally, using baseline 2000 SDI for classification may not fully capture the dynamic socioeconomic transitions in some provinces over the 21 years.

## Conclusions

Indonesia’s preventable AF burden is rising and varies by SDI. Developed regions need upstream metabolic risk reduction (eg, obesity and DM control), whereas less-developed regions require urgent mortality-focused actions through stronger primary care and improved hypertension management. SDI-targeted strategies are essential for matching interventions to local risk profiles.

## Funding Support and Author Disclosures

The authors have reported that they have no relationships relevant to the contents of this paper to disclose.
